# Clinically Relevant Anticancer Polymer Paclitaxel Therapeutics

**DOI:** 10.3390/cancers3010017

**Published:** 2010-12-23

**Authors:** Danbo Yang, Lei Yu, Sang Van

**Affiliations:** 1 Biomedical Engineering and Technology Institute, Institutes for Advanced Interdisciplinary Research, East China Normal University, 3663 North Zhongshan Road, Shanghai, 200062, China; E-Mail: dbyang@nbic.ecnu.edu.cn (D.Y.); 2 Biomedical Group, Nitto Denko Technical Corporation, 501 Via Del Monte, Oceanside, CA 92058, USA; E-Mail: sang_van@gg.nitto.co.jp (S.V.)

**Keywords:** polymer paclitaxel therapeutics, polymeric micelles, polymer paclitaxel conjugates

## Abstract

The concept of utilizing polymers in drug delivery has been extensively explored for improving the therapeutic index of small molecule drugs. In general, polymers can be used as polymer-drug conjugates or polymeric micelles. Each unique application mandates its own chemistry and controlled release of active drugs. Each polymer exhibits its own intrinsic issues providing the advantage of flexibility. However, none have as yet been approved by the U.S. Food and Drug Administration. General aspects of polymer and nano-particle therapeutics have been reviewed. Here we focus this review on specific clinically relevant anticancer polymer paclitaxel therapeutics. We emphasize their chemistry and formulation, *in vitro* activity on some human cancer cell lines, plasma pharmacokinetics and tumor accumulation, *in vivo* efficacy, and clinical outcomes. Furthermore, we include a short review of our recent developments of a novel poly(l-γ-glutamylglutamine)-paclitaxel nano-conjugate (PGG-PTX). PGG-PTX has its own unique property of forming nano-particles. It has also been shown to possess a favorable profile of pharmacokinetics and to exhibit efficacious potency. This review might shed light on designing new and better polymer paclitaxel therapeutics for potential anticancer applications in the clinic.

## 1. Introduction

Paclitaxel, extracted from the bark of the Pacific Yew tree [1], is one of the most effective anticancer agents known for the treatment of ovarian cancer, breast cancer, and lung cancer. Currently, commercial scale of paclitaxol bulk was produced from the cell line of *Taxus chinensis* [2]. Paclitaxel, however, suffers from poor bio-availability due to its low aqueous solubility. One approach to improve its solubility is to formulate paclitaxel with a mixture of cremophor and dehydrated ethanol [3]. A concern of solubility of paclitaxel was solved by employing the Cremophor/ethanol formulation, which led to commercialization of paclitaxel as Taxol®; however, the low therapeutic index of paclitaxel still persists due to the inability to selectively target tumor tissues and side-effects of Cremophor/ethanol diluent [4]. A number of efforts attempted to derivatize paclitaxel into small molecular water-soluble pro-drugs [5-7] but have not been pursued toward clinical developments.

**Figure 1.**
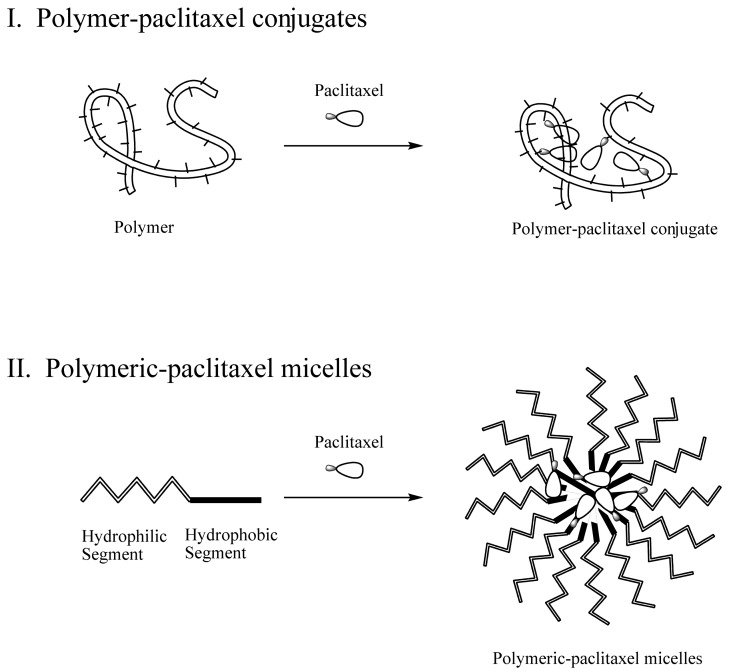
Schematic representation of polymer-paclitaxel conjugates (**I**) and polymeric-paclitaxel micelles (**II**).

Innovative strategies for solubilizing paclitaxel and targeting tumor tissues have also been actively pursued. One of the strategies is utilizing a polymer. Polymers can be used for solubilization in either covalent conjugation or non-covalent formulation of paclitaxel, namely polymer-paclitaxel conjugates or polymeric paclitaxel micelles, respectively (Figure 1). Reviews of polymer therapeutics were reported [8-10]; however, the topic was presented in aspects of general developments and advancements. Here, we provide a comprehensive, in-depth review of current clinically relevant polymer-paclitaxel therapeutics from design and chemistry to *in vitro/vivo* studies, and clinical outcomes. In addition, we present our recent preclinical polymer-paclitaxel nano-conjugate. We hoped that the in-depth and systematic review of the clinically relevant anticancer polymer-paclitaxel therapeutics would help us gain deeper understanding of this topic.

## 2. Polymer-Paclitaxel Conjugates

### 2.1. Design and Chemistry

**Figure 2.**
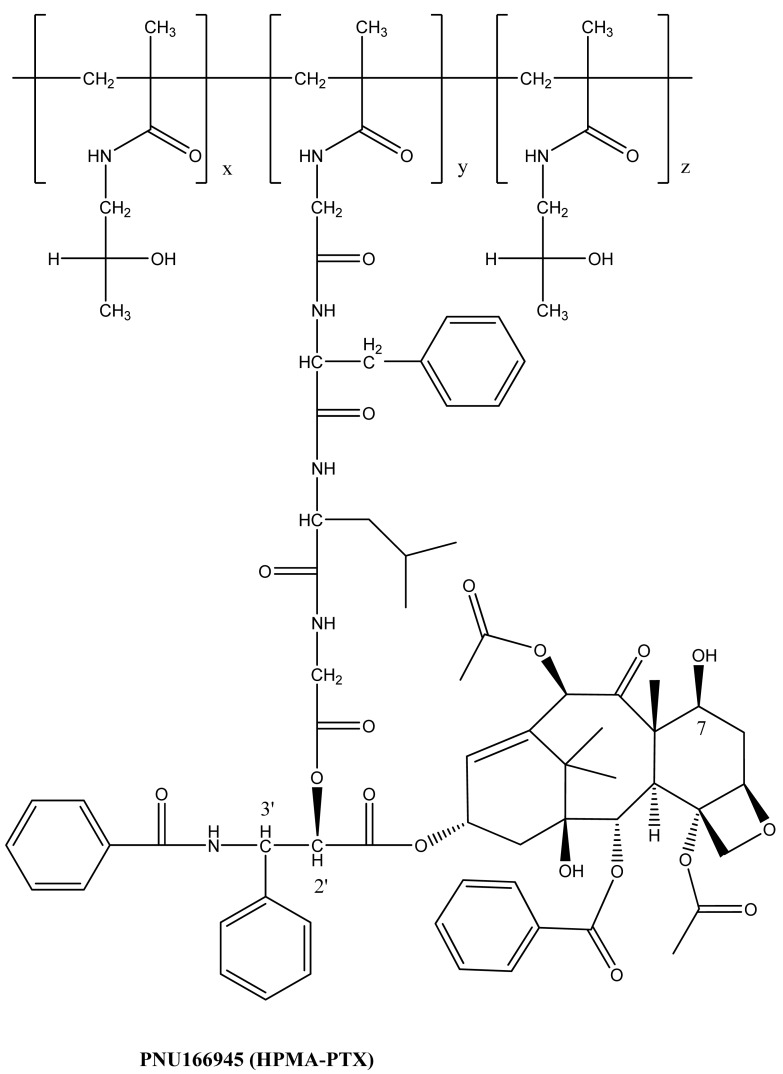
Structure of HPMA-PTX.

**Figure 3.**
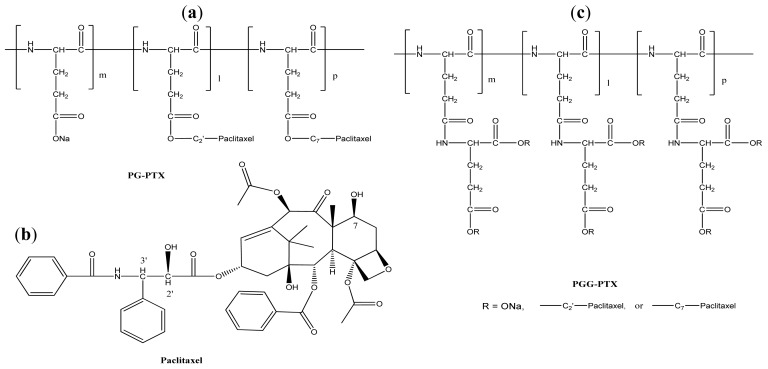
Structure of PG-PTX (**a**), paclitaxel (**b**), and PGG-PTX (**c**).

The concepts of coupling an anti-cancer drug to a polymer were essentially developed in the early 1980s [8,11]. It took about 20 years for the first polymer-paclitaxel conjugate (PNU166945) to enter a Phase I clinical trial [12]. Polymer-paclitaxel conjugates have been designed to improve the plasma pharmacokinetics by avoiding kidney filtration and to passively target hypervasculature, defective vascular architecture, and an impaired lymphatic drainage of tumor tissues, that is known as “enhanced permeability and retention” effects [13]. Recent clinical advances in polymer-paclitaxel conjugates are credited to co-polymer hydroxypropylmethacrylamide-paclitaxel conjugate (HPMA-PTX) [12] and poly(l-glutamic acid)-paclitaxel conjugate (PG-PTX) [14]. A schematic representation of the chemical structure of HPMA-PTX and PG-PTX is shown in Figure 2 and Figure 3a, respectively. HPMA-PTX is a water-soluble co-polymer in which paclitaxel is covalently bound through an ester bond at its 2′-OH position with an enzymatic degradable linker of Gly-Phe-Leu-Gly peptide. The polymer:paclitaxel ratio was approximately 19:1 (5%) by weight to weight. To improve paclitaxel drug loading, Li *et al.* [15] changed the polymer backbone to poly(l-glutamic acid). The amount of paclitaxel loading of PG-PTX improved to 20% by weight to weight, but the resulting conjugate contained mixed paclitaxel substitutions at both the C-2′ and C-7 ester positions [15]. With optimization of coupling chemistry of paclitaxel, paclitaxel loading increased to 37% by weight by weight [16]. In a similar platform, poly(lL-γ-glutamylglutamine)-paclitaxel nanoconjugate (PGG-PTX, as shown in Figure 3 (c)), was reported that with an additional glutamic acid as a linker between poly(l-glutamic acid) and paclitaxel, the paclitaxel drug loading was 35% weight by weight, and dissolution of PGG-PTX was faster than that of PG-PTX [17]. Futhermore, the glutamic acid linker provided enough flexibility of the PGG-PTX for self-assembly into nanoparticles whose size remains in the range of 12–15 nm (volume) over the concentration range of 25 to 2,000 μg/mL in saline [17]. Conjugation of paclitaxel can be achieved quantitatively, but it requires highly dried conditions to facilitate the completion of ester coupling in the presence of a 4-dimethylaminopyridine catalyst.

### 2.2. In Vitro Evaluation

*In vitro* evaluations of polymer-paclitaxel conjugates have been barely reported, yet polymer-paclitaxel conjugates are less cytotoxicity than that of free paclitaxel against many cancer cell lines [17-19]. Zou *et al.* [19] reported that a high dose of 1 mmol/L of PG-PTX could not achieve an IC_50_ value in H-460 cancer cell lines after 24 hours of drug exposure. IC_50_ value was determined to be 300–1000 nmol/L and 30–100 nmol/L, as paclitaxel equivalents, for PG-PTX after 48 hours and 72–96 hours of drug exposure, respectively, *versus* 10–30 nmol/L and 3–10 nmol/L for paclitaxel [19]. Van *et al.* [17] reported that IC_50_ values in human lung H-460 cancer cell lines were 2.31 μM and 2.25 μM, as paclitaxel equivalents, for PG-PTX and PGG-PTX after 72 hours of drug exposure, respectively, *versus* 0.15 μM for paclitaxel. The less cytotoxicity of the polymer-paclitaxel conjugates might be a key property to improve their paclitaxel-equivalent dose while maintaining a reasonable toxicity profile for *in vivo* animal models and clinical developments of chemotherapy *versus* that of Cremophor and ethanol formulation of paclitaxel.

### 2.3. Plasma Pharmacokinetics and Tumor Accumulation in Mouse Models

Polymer-paclitaxel conjugates are expected to prolong their plasma half-life and to have high tumor accumulation due to their slow excretion from kidney and the enhanced permeation and retention effects, respectively. Table 1 shows a comparison of pharmacokinetics (PK) and tumor accumulation of polymer-paclitaxel conjugates with paclitaxel. Li *et al.* [20] reported that PG-PTX prolonged over 100 times in plasma compared with that of Taxol, and tumor accumulation of PG-PTX was five times higher than that of Taxol. Similar trends were observed with PGG-PTX [21]. PK showed 23 times extended duration of PGG-PTX than that of Taxol, and tumor accumulation of PGG-PTX was seven times higher than that of Taxol [21]. On the basis of the results of PK and tumor accumulation, the findings of prolonged PK and enhanced tumor accumulation of polymer-paclitaxel conjugates could have important efficacy of antitumor activity.

### 2.4. In Vivo Efficacy

Polymer-paclitaxel conjugates exhibit superior antitumor activity, and *in vivo* efficacy results of the conjugates have been substantially reported [15,18-20,22,23]. Table 2 shows a summary of pre-clinical efficacy of PGG-PTX, Abraxane (albumin-bound paclitaxel), PG-PTX, and paclitaxel. PGG-PTX demonstrated significant antitumor activity in a well-defined dose-dependent manner with mice-bearing human lung H460 cancer cells, and the conjugate out-performed Abraxane in a number of mouse models [22]. PG-PTX also exhibited significant tumor growth delay after a single *i.v.* injection at 80 mg/kg (as paclitaxel equivalents) compared with that of paclitaxel in mice bearing murine ovarian OCA-1 carcinoma for two months [15]. Complete tumor regression with a single *i.v.* dose at 20 mg/kg, as paclitaxel equivalents, of PG-PTX was observed in rat bearing mammary 13762F adenocarcinoma [15]. In addition, mice bearing murine mammary MCa-4, MCa-35, and hepatocellular HCa-1, and soft-tissue Fsa-II cancer showed significant tumor growth delay at 120–160 mg/kg, as paclitaxel equivalents, of PG-PTX (as paclitaxel equivalents) [20]. Mice bearing human ovarian SKOV3ip1 and human breast MDA-MB-435Lung2 cancer had extended survival time and induced tumor regression in 50% of mice at the dose of 120 mg/kg, as paclitaxel equivalents, of PG-PTX [20]. Polymer-paclitaxel conjugates demonstrate excellent tumor growth delays and complete regression in many mouse models.

## 3. Polymeric-Paclitaxel Micelles

### 3.1. Design and Formulation

**Figure 4.**
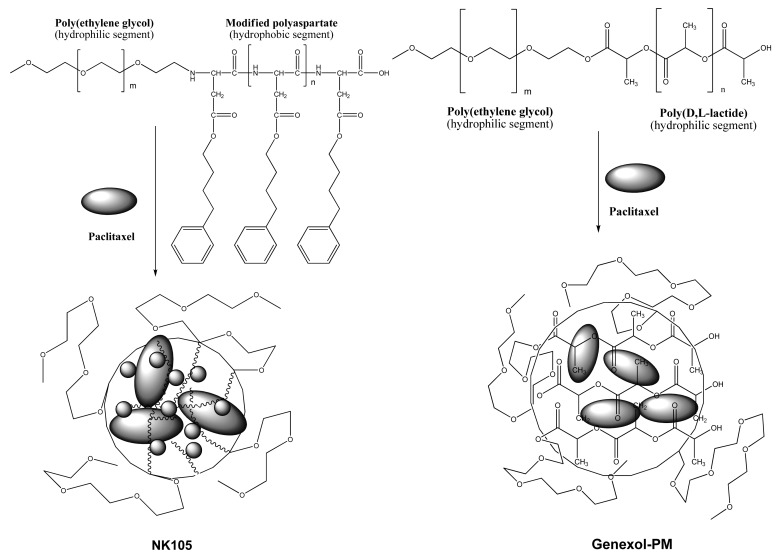
Schematic representation of NK105 and Genexol-PM.

Polymeric micelles are another effective formulation of paclitaxel. Two formulations of polymeric paclitaxel micelles advanced toward clinical developments are NK105 [24] and Genexol-PM [25], and their schematic representation is shown in Figure 4. The polymeric paclitaxel micelles are designed with diblock copolymers featuring hydrophilic segment of polyethylene glycol and hydrophobic portion of modified polyaspartate and poly(D, L-lactide) corresponding to NK105 and Genexol-PM, respectively. The hydrophobic core of the polymeric micelles can entrap free paclitaxel by hydrophobic-hydrophobic interactions. To increase efficiency of paclitaxel entrapment, 4-phenyl-1-butanol was chosen as modified polyaspartate groups after a series of candidate substances was screened [24]. Molecular weight of the NK105 was about 20,000 daltons, which comprises 8,000-dalton polyethylene glycol and 12,000-dalton modified polyaspartate [24]. Genexol-PM comprises of 2,000-dalton monomethoxy poly(ethylene) glycol and 1750-dalton poly(d,l-lactide) [25]. Both polymeric paclitaxel micelles formed stable nano-particles.

### 3.2. In Vitro Evaluation

In contrast to polymer-paclitaxel conjugates, polymeric paclitaxel micelles exhibit high *in vitro* cytotoxicity. The polymeric micelles show comparable *in vitro* cytotoxicity *versus* that of paclitaxel, against various human cancer cell lines. A summary of their *in vitro* cytotoxicity is shown in Table 3. Hamaguchi *et al.* [24] reported that NK105 and paclitaxel were tested on 12 human tumor cell lines derived from lung, gastric, esophagus, colon, breast and ovarian tumors, and the *in vitro* IC50 values of NK105 and paclitaxel showed similar cytotoxicity activity after 48 and 72 hours of drug exposure. Similar results of *in vitro* IC_70_ values of Genexol-PM and paclitaxel were observed with human breast MCF-7 and ovarian OVCAR-3 cancer cell lines [25]. The high cytotoxicity of polymeric paclitaxel micelles indicates that their active drug releases at different rates into the cells compared with the slow drug release of polymer paclitaxel conjugates.

### 3.3. Plasma Pharmacokinetics and Tumor Accumulation in Mouse Models

Interestingly, different polymeric paclitaxel micelles possess different plasma pharmacokinetics and tumor accumulation in mouse models compared with that of paclitaxel. Table 4 shows a summary of plasma pharmacokinetics and tumor accumulation of NK105, Genexol-PM, and paclitaxel in mouse models. Hamaguchi *et al.* [24] reported that when a single *i.v.* injection of the dose of 50 mg/kg (paclitaxel equivalents) of NK105 and paclitaxel in Colon 26-bearing CDF1 mice was administered, the plasma area under the curve (AUC) of NK105 was 86-fold higher than that of paclitaxel, and at the dose of 100 mg/kg (paclitaxel equivalents) AUC of NK105 was 50-fold higher than that of paclitaxel. The tumor accumulation AUC of NK105 was 24-fold higher than that of paclitaxel at the two doses of 50 mg/kg and 100 mg/kg. However, plasma AUC of Genexol-PM at the dose of 50 mg/kg paclitaxel equivalents was only comparable with that of paclitaxel at the dose of 20 mg/kg [25] in mice-bearing B16 melanoma tumors. Tumor accumulation AUC of Genexol-PM was 1.7-fold higher than that of paclitaxel. The differences in pharmacokinetics and tumor accumulation of the polymeric paclitaxel micelles might result from the different rate of clearance of the polymeric paclitaxel micelles in plasma and from different tumor models.

### 3.4. In Vivo Efficacy

Unlike the similarity of *in vitro* cytotoxicity of polymeric paclitaxel micelles, *in vivo* antitumor activity of polymeric paclitaxel micelles was superior compared with that of paclitaxel. A summary of results of polymeric paclitaxel micelle *in vivo* efficacy is shown in Table 5. Hamaguchi *et al.* [24] reported that the antitumor activity of NK105 administered at a 25 mg/kg dose, as paclitaxel equivalents, was comparable to that of paclitaxel which was administered at a 100 mg/kg dose. Furthermore, tumor suppression by NK105 increased in a dose-dependent manner, and tumor-free mice were observed at the dose of 100 mg/kg, as paclitaxel equivalents, of NK105. In addition, at the same doses, NK105 had better toxicity compared with paclitaxel by showing less weight loss and fewer degenerative myelinated fibres [24]. Superior *in vivo* efficacy was also reported with Genexol-PM compared with that of Taxol [25]. Tumor growth on nu/nu athymic mice bearing human ovarian SKOV-3 cancer was delayed after the treatment of Taxol at the maximum tolerated dose (MTD) of 20 mg/kg, up to 48 days but re-grew rapidly after that time. In contrast, after the treatment of Genexol-PM at the MTD dose of 60 mg/kg, tumor growth suppressed even after 70 days post treatment, and some mice experienced complete regression. Rac:Cr:(NCr)-nu athymic mice-bearing human breast MX-1 tumor treated with Genexol-PM were tumor-free after one month of treatment [25]. The encouraging *in vivo* efficacy results could have significant implications for future clinical developments.

## 4. Clinical Outcomes of Polymer-Paclitaxel Therapy

### 4.1. Phase I Pharmacokinetics and Toxicity of Polymer Paclitaxel Therapy

Favorable results of *in vivo* efficacy, pharmacokinetics, and tumor accumulation of polymer paclitaxel therapeutics in animal models, provide great insights of the innovative drug delivery systems. Nevertheless, Phase I pharmacokinetic and toxicity studies determine the feasibility of a drug development program. Tables 6–9 show results of Phase I pharmacokinetics and toxicity of the clinical trials of polymer paclitaxel therapeutics up to date. Meerum Terwogt *et al.* [12] reported that dose-limiting toxicity of PNU166945 (HPMA-PTX) was not observed at the studies dose levels of 80 mg/m^2^ even up to 196 mg/m^2^, as paclitaxel equivalents; therefore, the maximum tolerated dose (MTD) was not reached. The plasma AUC of HPMA-PTX increased from 318 to 450 h·μM corresponding to the starting dose of 80 mg/m^2^ and escalating to 196 mg/m^2^. The concentration C_max_ also increased from 40 to 75 μM, corresponding with the doses. The t_1/2_ and total clearance was about 6.0 to 6.5 h and from 0.5 to 0.9 L/h, respectively, for all the doses. However, the trial study was discontinued due to severe neurotoxicity observed in rat studies [12].

Pharmacokinetics (PK) of PG-PTX showed that its half-life was prolonged [14]. The PK was determined during the first course of treatment and at 24 and 48 hours during the second course [14], which was different and the results could not be used to compare with the results of the normal PK samplings of the first course [12,26,27]. Results of Phase I pharmacokinetic study and toxicity of PG-PTX are presented in Table 7. In the study, dose limiting toxicity of neutropenia was observed at 266 mg/m^2^ (paclitaxel equivalents) in Phase Ia, and the MTD was determined to be 233 mg/m^2^. With the MTD and 3-weekly treatment cycle (Phase Ia), two patients experienced neutrophils toxicity and two patients experienced white blood cell counts (WBC) toxicity Grade 3 or greater. More WBC and neutrophils toxicity was observed with 2-weekly treatment cycle at doses of 177 and 210 mg/m^2^, paclitaxel equivalents [14]. Other non-hematological toxicities Grade 3 or greater such as neuropathy-sensory and motor, liver disfunction, and diarrhea were also observed in the patients with the 2-weekly treatment cycle. Plasma AUC of PG-PTX increased as the doses increased, except one patient treated with a dose of 88 mg/m^2^. At the MTD and 3-weekly treatment, the plasma AUC, t_1/2_, and total clearance of PG-PTX was 2326 h-μM, 120 h, and 0.28 L/h, respectively. The results from the Phase I study of PG-PTX show that polymer-paclitaxel conjugate prolonged its half-life with limited volume of distribution.

Polymeric paclitaxel micelles were investigated in a Phase I trial and showed that they were well tolerated and extended blood circulation [26,27,32]. Results of Phase I pharmacokinetics and toxicity of Genexol-PM and NK105 are shown in Tables 8 and 9, respectively. On a 3-weekly regimen for patients with advanced malignancies, the paclitaxel AUC and peak concentration of Genexol-PM increased linearly with escalating dose, except at 230 mg/m^2^. The dose-limiting toxicity effects of neuropathy-sensory and motor and neutropenia were observed at a dose of 390 mg/m^2^. Paclitaxel AUC and peak concentration of NK105 increased as the doses increased from 10 to 180 mg/m^2^ [27]. Surprisingly, the paclitaxel AUC and peak concentration of NK105 [27] were exceptionally high compared with those of Taxol [33] and Genexol-PM [26]. The paclitaxel AUC of NK105 and peak concentration was 110 to 532 h·μM and 10 to 53 μM, respectively, corresponding to the doses of 40 mg/m^2^ to 180 mg/m^2^, whereas the paclitaxel AUC and peak concentration of paclitaxel was 22 h·μM and 3 μM [33], respectively. However, at 180 mg/m^2^, as paclitaxel equivalents, of NK105, two out of five and three out of five patients acquired neutropenia grade 4 and grade 3, respectively. Plasma AUC of NK105 at 150 mg/m^2^ was about 24-fold higher than that of paclitaxel at 175 mg/m^2^. Both polymeric paclitaxel micelles seem to be well tolerated.

### 4.2. Clinical Outcomes of PG-PTX and Paclitaxel Therapy in Patients with Recurrent Epithelial Ovarian, Fallopian Tube, Primary Peritoneal, or Metastatic Castration-Resistant Prostate Cancer

Paclitaxel and platinum-based chemotherapy is the standard regimen for recurrent epithelial ovarian cancer [31]. With favorable preclinical [23] and clinical [14] pharmacokinetics and establishment of MTD of 235 mg/m^2^ (paclitaxel equivalents), PG-PTX was investigated for Phase II toxicity, response rate, and time to disease progression (TTP) in women with recurrent epithelial ovarian, primary peritoneal, or fallopian tube carcinoma in multi-centers and multi-trials [28,29]. Clinical outcomes of the PG-PTX Phase II therapy are shown in Table 10. Response rate and median TTP of PG-PTX were 10% and 2.1 months, respectively, which were reported in one Phase II study [28], and response rate and median progression-free survival for PG-PTX were 16% and 2.8 months, respectively, which were reported in another Phase II study [29]. Those response rates and the median progression-free survival numbers were not favorable compared with that of the standard regimen of paclitaxel and platinum-based chemotherapy which exceeded 70% of response rate [28,34] and 18–30 months of median progression-free survival [31,34]. Furthermore, a Phase II study of PG-PTX in combination with transdermal estradiol for the treatment of metastatic castration-resistant prostate cancer after docetaxel chemotherapy was also not positive due to no responses in measurable diseases, the 0.9-month median TTP, and the 7.8-month median overall survival [30], compared with 12% response rate and 16.5-month median overall survival for the docetaxel plus prednisone treatment [35] and with 17% response rate, 17.5-month median overall survival, and 6.3-month median TTP of the docetaxel and estramustine regimen [36]. Overall, the response rate, median overall survival, and median TTP of PG-PTX in the multi-phase II trials were not improved compared with those of standard chemotherapy.

### 4.3. Clinical Outcomes of Polymer Paclitaxel and Paclitaxel Therapy in Patients with Metastatic Breast Cancer

Paclitaxel was approved for treatment in patients with metastatic breast cancer with typical response rate of 25% [37] and median overall survival of 13–20 months [37-39]. The multi-center Phase II trials of Genexol-PM and PG-PTX were carried to evaluate their response rate, median TTP, and median overall survival. Table 11 presents a summary of clinical outcomes of polymer paclitaxel therapy in patients with metastatic breast cancer. Lin *et al.* [40] reported that Phase II study of PG-PTX resulted in unexpected incidence of hypersensitivity reactions and neurotoxicity at a dose of 175 mg/m^2^ with 3-weekly cycle regimen. The objective responses were observed in four of 18 patients (22% of overall response rate). The study was terminated early due to higher-than-expected rate of hypersensitivity reactions. However, polymeric paclitaxel micelle Genexol-PM showed surprisingly 59% of response rate and 9-month median TTP [41], compared to 25% of response rate and 3-5 months of median TTP of paclitaxel [37,38]. On the basis of response rate and median TTP, Genexol-PM seems to be an encouraging new formulation of paclitaxel for treatment of metastatic breast cancer.

### 4.4. Clinical Outcomes of Polymer Paclitaxel and Paclitaxel Therapy in Patients with Non-Small-Cell Lung Cancer

A combination of paclitaxel and platinum-based chemotherapy seems to be a choice for treatment of patients with non-small-cell lung cancer (NSCLC) [39-42]. A single agent PG-PTX [43,44] and a combination of PG-PTX and carboplatin [45] in patients with advanced NSCLC were investigated to evaluate the survival as the primary study end point, and the response rate, time to progress (TTP), safety, and quality of life as secondary objectives. Clinical outcomes of PPX therapy of three Phase III trials are shown in Table 12. O'Brien *et al.* [43] reported that the overall survival was similar between chemotherapy-naive PS 2 patients with advanced NSCLC who randomly received single-agent PPX at 175 mg/m^2^ or a standard single agent vinorelbine or gemcitabine. Median survival and 1-year survival were 7.3 months and 26%, respectively, for PG-PTX arm, *versus* 6.6 months and 26% for the standard control arm which was statistically non-significant. The patients treated with PG-PTX experienced 30% of neuropathy, whereas, with the standard control treatment, experienced 5% of neuropathy. In a different Phase III trial [44], results showed that median and 1-year survival were 6.9 months and 25%, respectively, for the PG-PTX arm (175 mg/m^2^ or 210 mg/m^2^) *versus* 6.9 months and 29% for the docetaxel arm (75 mg/m^2^). Due to adverse effects, more patients treated with PG-PTX discontinued their treatment. Langer *et al.* [45] reported that the overall survival was also similar between chemotherapy-naive PS 2 patients with advanced NSCLC who randomly received carboplatin at AUC 6 and either PG-PTX at 210 mg/m^2^ or paclitaxel 225 mg/m^2^. Median overall survival was 7.8 months for PG-PTX arm *versus* 7.9 months for paclitaxel, and 1-year survival was 31% for both arms. A combination of polymeric paclitaxel micelle Genexol-PM with cisplatin was also investigated in a Phase II trial [46]. Results of the Phase II study in patients with advanced NSCLC are shown in Table 12. Median overall survival and 1-year survival was 21.7 months and 60% for Genexol-PM at 230 mg/m^2^ and cisplatin at 60 mg/m^2^. Overall, a combination of Genexol-PM with cisplatin chemotherapy demonstrated significant antitumor activity, compared with a single agent PG-PTX, a combination of PG-PTX with carboplatin, or paclitaxel with platinum-based chemotherapy.

### 4.5. Clinical Outcomes of Polymer Paclitaxel and Paclitaxel Therapy in Patients with Advanced Gastric Cancer

The incidence of gastric cancer is very high in Asia, particularly in Japan, Korea, and China [47]. 5-Fluorouracil and/or cisplatin-based combination chemotherapy is widely used for the treatment of gastric cancer but only achieves modest benefits [48,49]. Paclitaxel and a combination of paclitaxel with platinum-based chemotherapy were investigated for better treatment of this disease. Results showed that paclitaxel and its combination with paclitaxel delivered 23, 33, and 43% of response rate and 43, 23, and 40 months of 1-year survival with treatment of paclitaxel [50], paclitaxel plus carboplatin [48], and paclitaxel plus cisplatin [49], respectively. Polymeric paclitaxel micelles Genexol-PM [51] and NK105 [52] were also evaluated for efficacy and safety as Phase II trials. Results of the trials are shown in Table 13. Response rate and median overall survival for NK105 were 25% and 10.2 months, respectively. A combination of Genexol-PM with cisplatin improved up to 46% of response rate and 13.8 months of median overall survival, compared with 33% response rate and 7.5 months of median overall survival for paclitaxel plus caroplatin [48] and 43% response rate and 11.2 months of median overall survival for paclitaxel plus cisplatin [49]. Overall, polymeric paclitaxel micelles provided some benefits to patients with advanced gastric cancer in terms of response rate, median overall survival, and 1-year survival.

### 4.6. Advantages and Disadvantages of Each Formulation and Perspective of Polymer Paclitaxel Therapeutics for Future Clinical Applications

Polymer paclitaxel conjugates and encapsulates exhibit their unique advantages and face their own intrinsic issues. Polymer paclitaxel conjugates gain ground on: (1) robust conjugation chemistry; (2) ease of large scale manufacture, and (3) easy formulation. To the best of our knowledge, with anhydrous dimethylformamide as a solvent and trace of 4-dimethyl-aminopyridine as a catalyst, coupling chemistry of paclitaxel onto a polymer with a pendent carboxylic acid is relatively simple, robust, and quantitative [17]. Lab-scales of 10-to 100-g batches of lyophilized polymer-paclitaxel conjugate can be delivered within a few days using tangential flow filtration. The lyophilized polymer-paclitaxel conjugate as an active pharmaceutical ingredient can be reconstituted in water or physiological saline before injection [18,22]. The current issues with polymer-paclitaxel conjugates are: (1) broad polydispersity indices (PDI); (2) randomness of conjugation; and (3) mixed paclitaxel substitutions at both the C-2′ and C-7 ester positions at γ-position of a pendent carboxylic acid of a poly(L-glutamic acid) (PG) [15] as shown in Figure 3 (a) and γ-and β-positions of a pendent carboxylic acid of a poly(L-γ-glutamylglutamine) [17]. Typically, PG is obtained from a chemical supplier with broad PDIs of 1.5–1.8. The randomness of conjugation and the mixed substitutions of paclitaxel provide an unclear picture of paclitaxel release. These disadvantages may be key obstacles in chemistry, manufacture, and control of clinical products. Furthermore, when developing a novel polymer-paclitaxel conjugate, chemistry design should be focused on evading liver, lung, kidney, and spleen uptakes to improve its efficacy. If one provided mono-disperse polymers with precise chemical conjugation and ease of modification of multifunctional groups, a polymer drug conjugate field would be clearly and rapidly advanced. Polymer paclitaxel micelles receive attention based on: (1) ease of small (<10-g) scale formulation of paclitaxel; and (2) complete controlled release of paclitaxel. However, when one carries out scaled-up formulations (10-and 100-g batches) of polymeric paclitaxel micelles, their manufacture process and quality control may face some issues. A key parameter that must be overcome to deliver quality clinical materials may be to demonstrate a robust processing of large scale production of the micelle particles. With regard to poor correlation between preclinical and clinical outputs, we speculate that mouse models were not comparable to human cancer diseases. We also believe that when polymer paclitaxel formulations were injected in mice, less attention was paid to hypersensitivity reactions. As a result, an unexpected incidence of hypersensitivity reactions was observed in clinical trial study [40]. Combining polymer-paclitaxel conjugates with polymeric paclitaxel micelles would provide an innovative approach for polymer paclitaxel therapeutics.

## 5. Conclusions

Polymer therapeutics is an innovative formulation of paclitaxel. Polymer paclitaxel therapeutics includes covalent conjugated paclitaxel and non-covalent micelle encapsulated paclitaxel. *In vitro* cytotoxicity of polymeric paclitaxel micelles was similar to paclitaxel, whereas *in vitro* IC_50_ values of polymer paclitaxel conjugates were much less than that of paclitaxel. Plasma pharmacokinetics of polymeric paclitaxel micelle Genexol-PM in animal models was not much different compared with that of paclitaxel. However, plasma half-life of polymeric paclitaxel micelle NK105 exhibited 50 to 86-fold higher than that of paclitaxel, and tumor accumulation of NK105 was 24-fold higher than that of paclitaxel. Both polymer-paclitaxel conjugates and polymeric paclitaxel micelles showed major differences in *in vitro* cytotoxicity, pharmacokinetics (PK), and tumor accumulation from each other. However, both formulations of paclitaxel delivered higher MTD as paclitaxel equivalents and demonstrated significant antitumor activity in animal models. Phase I study of HPMA-PTX was terminated early due to neurotoxicity. The results of Phase III of PG-PTX indicated that response rate and survival were not improved compared with the standard chemotherapy in patients with metastatic prostate, breast, and lung cancer PG-PTX. However, polymeric paclitaxel micelles demonstrated significant antitumor activity in patients with metastatic breast, lung, and gastric cancer. To confirm the survival benefits of treatment with polymeric paclitaxel micelles, a large Phase III trial should be conducted.

## Figures and Tables

**Table 1.** Plasma pharmacokinetics and tumor accumulation of polymer-paclitaxel conjugates and paclitaxel in mouse models.

**Mice**	**bearing**	**Treatment**	**Dose (mg/kg)**	**AUC (μg·h/g tissue)**		**Ref.**
				**plasma**	**tumor**	
Female C3Hf/Kam	Murine ovarian Oca-1 carcinoma	PG-PTX	20	184.1	1097.3	[23]
		Paclitaxel	20	1.8	211.4	
*PG-PZX*/Paclitaxel ratio				100.9	5.2	
Female nude	Human H460 lung cancer	PGG-PTX	40	3454.4	2496	[21]
		Paclitaxel	40	146.3	322.5	
*PG-PTX*/Paclitaxel ratio				23.6	7.7	

**Table 2.** Results of *in vivo* efficacy of polymer-paclitaxel conjugates.

**Mice/Rats**	**Bearing cancer cells**	**Treatment**	**Administration**	**Dose (mg/kg)**	**Efficacy**	**Toxicity**	**Ref.**
Female BALB/c nude mice	Human lung H-460 cancer	PGG-PTX	a single *i.p.* dose	150	Significant antitumor activity in a well-defined dose-dependent manner.	Increased in toxicity as the doses increased	[22]
				200			
				250			
				300			
	Human lung H-460 cancer	PGG-PTX	a single *i.p* dose	300	Significant inhibition of tumor relative to that in the control mice (P = 0.001) but not to that in the Abraxane treated mice (P = 0.92).	Both drugs produced equivalent acute reductions in body weight. Weight recovery more rapid with Abraxane.	
		Abraxane		250			
	Human 2008 ovarian carcinoma	PGG-PTX	a single *i.p* dose	300	Significant inhibition of tumor relative to that in the control mice (P = 0.006) and that in the Abraxane treated mice (P = 0.025).	Both drugs produced equivalent acute reductions in body weight. Weight recovery more rapid with Abraxane.	
		Abraxane		200			
Female BALB/c nude mice	Murine B16 melanoma	PGG-PTX	a single *i.p* dose	350	Significant inhibition of tumor relative to that in the control mice (P = 0.0002) and that in the Abraxane treated mice (P = 0.020).	Both drugs produced equivalent acute reductions in body weight. Weight recovery more rapid with Abraxane.	[22]
		Abraxane		150			
	Human lung H-460 cancer	PGG-PTX	a single *i.v.* dose weekly for 3 weeks	140	Significant inhibition of tumor relative to that in the Abraxane treated mice (P = 0.020).	Mimimal weight loss with PGG- PTX but significant weight loss with Abraxane treatments.	
		Abraxane		40			
C3H/Kam mice	Murine ovarian OCA-1 carcinoma	PG-PTX	a single *i.v.* dose	80	Significant tumor growth delay at 80 mg/kg compared with paclitaxel. Tumor growth suppressed at 160 mg/kg. 25 of 26 mice remained tumor-free after 2 months.	No mice treated with PG-PTX died during the experimental period, whereas 2 of 13 mice treated with paclitaxel died.	[15]
				160			
		Paclitaxel		80			
Female Fischer 344 rats	Rat mammary 13762F adeno-carcinoma	PG-PTX	a single *i.v.* dose	20	Tumor suppression at 20 mg/kg of PG-PTX. Tumor regression at 40 mg/kg of PG-PTX.		
				40			
		Paclitaxel		40			
C3H/Kam mice	Murine mammary MCa-4	PG-PTX	a single *i.v.* dose	60	Tumor regression at 120 mg/kg from days 8-19, but tumors reappeared on day 21, with slower rate compared with Taxol treated mice.		[20]
				120			
		Paclitaxel		60			
	Murine mammary MCa-35	PG-PTX	a single *i.v.* dose	80	Significant tumor growth delay at the MTD of 160 mg/kg paclitaxel.		
				160			
		Paclitaxel		80			
C3H/Kam mice	Murine Hepatocellular HCa-1	PG-PTX	a single *i.v.* dose	80	Significant tumor growth delay at 160 mg/kg paclitaxel.	Mice treated with PG-PTX maintained their body weight; whereas mice treated with paclitaxel quickly lost weight.	[20]
				160			
		Paclitaxel		80			
	Murine soft-tissue Fsa-II sarcoma	PG-PTX	a single *i.v.* dose	80	Similar patterns of sensitivity to PG-PTX and paclitaxel.	Both PG-PTX and paclitaxel reduced body weight loss.	
				160			
		Paclitaxel		80			
		PG-PTX	Three injections	120	Extended the survival time but no statistically significant difference compared with paclitaxel-treated mice at 100 days. No difference even with treatment of 3 injections.	20-30% survival at 100 days in paclitaxel-treated mice and PG-PTX-treated mice	
Female BALB/c nude mice	Human ovarian SKOV3ip1 cancer	PG-PTX	a single *i.v.* dose	60			
				120			
		Paclitaxel		60			
	Human breast MDA-MB-435Lung2 breast cancer	PG-PTX	Three injections	60	At 120 mg/kg, PG-PTX induced tumor regression in 50% of animals. Similar antitumor activity between multiple injections and a single injection.		
		PG-PTX	A single *i.v.* injection	60			
				120			
		Paclitaxel		60			

**Table 3.** *In vitro* evaluations of polymeric-paclitaxel micelles and Taxol in various cancer cell lines.

**Cancer**	**Drug**	**Genexol-PM**	**Taxol**	**NK-105**	**Taxol**	**NK-105**	**Taxol**
		**IC_70_ (**μ**g/mL)**		**IC_50_ (**μ**M)**		**IC_50_ (**μ**M)**	
	**Tumor cell line**	**(96 h drug exposure)**		**(48 h drug exposure)**		**(72 h drug exposure)**	
**Eesophageal**	TE-1			>1.0	>1.0	0.01	0.02
	TE-8			0.02	0.02	0.01	0.01
**Lung**	PC-14			0.01	0.01	0.01	0.01
	PC-14/TXT			0.15	0.09	0.08	0.06
	H460					0.03	0.01
**Breast**	MCF-7	0.002	0.002	>1.0	>1.0	0.01	0.01
**Stomach**	MKN-28			0.03	0.03	0.01	0.21
	MKN-45			0.02	0.07	0.01	0.02
**Colon**	DLD-1			0.95	0.26	0.29	0.20
	HT-29			0.01	0.01	0.01	0.01
	HCT116					0.03	0.01
**Ovarian**	MCAS			0.01	0.01	0.01	0.01
	OVCAR-3	0.002	0.004	>1.0	>1.0	>1.0	>1.0
**Pancreatic**	AsPC-1					0.02	0.02
	PAN-9					0.03	0.02
	PAN-3					0.01	0.004
**Ref.**		[25]		[24]			

**Table 4.** A summary of *in vivo* efficacy of polymeric paclitaxel micelles and Taxol in mouse models.

**Mice/Rats**	**Bearing cancer cells**	**Treatment**	**Administration**	**Dose (mg/kg)**	**Efficacy**	**Toxicity**	**Reference**
Female BALB/c nude mice	Human colon HT-29 cancer	NK105	a single *i.v.* dose weekly for 3 weeks	25	Tumor suppression by both drugs increased in a dose-dependent manner. Superior antitumor acitivity compared with paclitaxel (P < 0.001). Tumor disappeared after the first dosing with NK105 at 100 mg/kg and all the mice remained tumor-free thereafter.	Less weight loss with NK105 compared with Taxol at the same given dose. Fewer degenerative myelinated fibers compared with paclitaxel (P < 0.001).	[24]
				50			
				100			
		Paclitaxel		25			
				50			
				100			
Female BALB/c nude mice	Human ovarian SKOV-3 cancer	Genexol-PM	a single *i.v.* dose on days 0, 4, and 8	60	Significant inhibition of tumor relative to that in the paclitaxel treated mice.	No mice treated with Genexol- PM died during the experimental period.	[25]
		Paclitaxel		20			
Tac:Cr:(NCr)-nu mice	Human breast MX-1 cancer	Genexol-PM	a single *i.v.* dose on days 0, 1, and 2	60	Significant inhibition of tumor relative to that in the paclitaxel treated mice. After 1 month, all the mice treated with Genexol-PM were tumor-free.	No mice treated with Genexol- PM died during the experimental period.	
		Paclitaxel		20			

**Table 5.** Plasma prightharmacokinetics and tumor accumulation of polymeric paclitaxel micelles and paclitaxel in mouse models.

				**AUC (μg·h/mL)**		
**Mice**	**Bearing**	**Treatment**	**Dose (mg/kg)**	**Tumor**	**Plasma**	**Ref.**
Female SPF C57BL/6	B16 melanoma	Genexol-PM	50	3714	77	[25]
		Paclitaxel	20	2140	85	
Genexol-PM/ Paclitaxel ratio				1.7	0.9	
Female CDF1	Colon 26	NK-105	50	3192	7862	
		Paclitaxel	50	133	91	
NK-105/Paclitaxel ratio				24.0	86.4	[24]
Female CDF1	Colon 26	NK-105	100	7965	15574	
		Paclitaxel	100	331	309	
NK-105/Paclitaxel ratio				24.1	50.4	

**Table 6.** Results of Phase I pharmacokinetic study and toxicity of HPMA-PTX in cancer patients [12].

**Pharmacokinetics of HPMA-PTX**					

**Pharmacokinetics of total paclitaxel^b^**					

**Dose (mg/m^2^)^a^**	**N**	**AUC(h·μM)**	**C**_**max**_**(μM)**	**t**_**1/2**_ **(h)**	**CL**_**total**_ **(L/h)**
80	3	318 ± 99	40.1 ± 5.1	6.5 ± 0.3	0.54 ± 0.22
100	3	268 ± 56	44.1 ± 10.1	5.7 ± 0.6	0.87 ± 0.21
140	3	413 ± 107	61.2 ± 9.3	6.5 ± 0.7	0.76 ± 0.19
196	3	450 ± 19	74.9 ± 4.3	6.6 ± 0.8	0.84 ± 0.96

**Toxicity of HPMA-PTX**					

**Dose (mg/m^2^)^a^**	**N**	**Grade 3 or greater**			

		**Hematological toxicities**			**Neuromuscular toxicities**

		**Anemia^c^**	**Neutropenia**	**granulo-cytopenia^d^**	**Peripheral neuropathy**
80	3	0	0	0	0
100	3	1	0	0	0
140	3	0	0	0	0
196	3	0	0	0	1

a As paclitaxel equivalents.

b Pharmacokinetics of polymer-drug micelles was determined during the first course.

c One more patient experienced anemia grade 3, but dose not specified.

d One patient experienced granulocytopenia grade 3, but dose not specified.

**Table 7.** Results of Phase I pharmacokinetic study and toxicity of PG-PTX in cancer patients [14].

**Pharmacokinetics of PG-PTX**					
		**Pharmacokinetics of conjugated paclitaxel^b^**			
**Dose (mg/m^2^)^a^**	**N**	**AUC (h·μM)**	**C**_**max**_**(μM)**	**t**_**1/2**_ **(h)**	**CL**_total_ **(L/h)**
***Phase la (3-weekly)***					
11	1	21	-	4.0	1.11
22	1	90	-	6.1	0.60
44	1	221	-	7.8	0.38
88	1	461	-	24.9	0.45
88	1	215	-	74.0	0.94
177	1	1052	-	145.0	0.41
233	4	1854± 670	-	120 ± 28	0.28 ± 0.06
266	4	2326 ± 1262	-	119 ± 15	0.35 ± 0.23
***Phase Ib (2-weekly)***					
177	5	937 ± 429	-	128 ± 72	0.46 ± 0.16
210	3	1309± 187	-	69 ± 47	0.35 ± 0.10

**Toxicity of PG-PTX**									
		**Grade 3 or greater**							
		**Hematogical toxicities**		**Non-hematological toxicities**					
**Dose (mg/m^2^)^a^**	**N**	**WBC**	**Neutrophils**	**Hypersensitivity**	**Neuropathy-motor**	**Neuropathy-sensory**	**Liverdysfunction**	**Stomatitis**	**Diarrhea**
***Phase la (3-weekly)***									
88	2	1	1	0	0	0	0	0	0
175^c^	1		1	0	0	0	0	0	0
177	1	0	0	1	0	0	0	0	0
233	7	2	2	0	0	0	0	0	0
266	6	4	4	1	1	0	1	2	0
***Phase Ib (2-weekly)***									
177	6	2	2	0	0	2	1	0	0
210	4	3	4	0	1	2	0	0	1

a As paclitaxel equivalents.

b Pharmacokinetics of polymer-bound paclitaxel was determined during the first course and at 24 and 48 hours during the second course.

c Patient with dose reduction from 233 mg/m^2^.

**Table 8.** Results of Phase I pharmacokinetic study and toxicity of Genexol-PM in cancer patients [26].

**Pharmacokinetics of Genexol-PM**					

**Pharmacokinetics of paclitaxel^b^**					

**Dose (mg/m^2^)^a^**	**N**	**AUC (h·μM)**	**C**_**max**_**(μM)**	**t**_**1/2**_ **(h)**	**CL**_**total**_ **(L/h/m^2^)**
135	3	6.4 ± 1.5	1.6 ± 0.3	12.7 ± 4.2	25.5 ± 5.3
175	3	6.7 ± 1.6	1.7 ± 0.2	12.5 ± 2.5	32.0 ± 8.8
230	6	22.8 ± 4.7	5.5 ± 1.8	11.0 ± 1.9	12.1 ± 2.5
300	6	13.6 ± 5.0	3.6 ± 1.7	11.4 ± 2.4	29.3 ± 13.8
390	3	32.2 ± 9.7	7.7 ± 1.3	17.9 ± 1.0	14.9 ± 4.5

**Toxicity of Genexol-PM**							

**Grade 3 or greater**							

**Dose (mg/m^2^)^a^**	**N**	**Hematological toxicities**		**Neuromuscular toxicities**			

		**Anemia**	**Neutropenia**	**Thrombocytopenia**	**Neuropathymotor**	**Neuropathysensory**	**Myalgia**
135	3	0	0	0	0	0	0
175	3	0	0	0	0	0	0
230	6	0	2	0	0	0	1
300	6	0	3	0	0	0	1
390	3	0	1	0	1	2	0

a As paclitaxel equivalents.

b Pharmacokinetics of polymer-drug micelles was determined during the first course.

**Table 9.** Results of Phase I pharmacokinetic study and toxicity of NK105 in cancer patients [27].

**Pharmacokinetics of NK105**					

**Pharmacokinetics of paclitaxel^b^**					

**Dose (mg/m^2^)^a^**	**N**	**AUC (h·μM)**	**C**_**max**_**(μM)**	**t**_**1/2**_ **(h)**	**CL**_**total**_ **(L/h/m^2^)**
10	1	13.3	1.1	9.0	0.88
20	1	34.1	3.4	8.5	0.69
40	1	110.0	10.4	13.2	0.43
80	1	174.8	21.6	7.0	0.54
110	3	271.7 ± 45.8	27.4 ± 6.6	9.7 ± 1.6	0.48 ± 0.08
150	7	433.0 ± 41.2	47.1 ± 6.5	10.6 ± 1.3	0.41 ± 0.04
180	4	532.2 ± 139.5	53.5 ± 21.8	11.3 ± 0.6	0.42 ± 0.10

**Toxicity of NK105**							

**Grade 3 or greater**							

		**Hematological toxicities**			**Neuromuscular toxicities**	

**Dose (mg/m^2^)^a^**	**N**	**Leukopenia**	**Neutropenia**	**Thrombocytopenia**	**Neuropathy**	**Myalgia**
10-110	7	2	2	0	0	0
150	7	2	5	0	0	0
180	5	3	5	0	0	0

a As paclitaxel equivalents;

b Pharmacokinetics of polymer-drug micelles was determined during the first course

**Table 10.** Clinical outcomes of PG-PTX and paclitaxel therapy in patients with metastatic castration-resistant prostate, recurrent epithelial ovarian, fallopian tube, or primary peritoneal carcinoma.

**Treatment**		**Response rate**	**Median TTP**	**Median OS**	**1-year survival**	**Ref.**

	**N**	**(%)**	**(months)**	**(months)**	**(%)**	
*Polymer-paclitaxel therapeutics*						
PG-PTX^a^	99	-	2.1	-	-	[28]
PG-PTX^b^	25	10	2.8*	15.4	-	[29]
PG-PTX/estradiol^c^	21	16	0.9	7.8	-	[30]
*Paclitaxel therapeutics*						
Paclitaxel^d^	101	-	30*	77	97	[31]

* The median progression-free survival (PFS);

a Patients with recurrent epithelial ovarian, fallopian tube, or primary peritoneal cancer, received CT-2103 (paclitaxel) at 175 mg/m^2^ *i.v.* infusion over period of 10 min, every 21 days;

b Patients with recurrent ovarian or primary peritoneal cancer, received PPX (paclitaxel) at 235 mg/m^2^ *i.v.* infusion over period of 10 min, every 21 days;

c Patients with metastatic castration-resistant prostate cancer, received transdermal estradiol (0.2 mg/24 h) for 4 weeks, and followed by the same dose of transdermal estradiol and PPX (paclitaxel) at 150 mg/m^2^ *i.v.* infusion over period of 10–20 min, every 28 days.

d Patients with epithelial ovarian, primary, or fallopian tube cancer, received paclitaxel at 175 mg/m^2^ *i.v.* infusion over period of 3 h, every 21 days, with hypersensitivity reaction to pre-medications.

**Table 11.** Clinical outcomes of polymer paclitaxel and paclitaxel therapy in patients with metastatic breast cancer.

**Treatment**		**Response rate**	**Median TTP**	**Median OS**	**1-year survival**	**Ref.**

	**N**	**(%)**	**(months)**	**(months)**	**(%)**	
*Polymer-paclitaxel Therapeutics*						
Genexol-PM^a^	41	59	9	-	-	[41]
PG-PTX^b^	18	22	-	-	-	[40]
*Paclitaxel Therapeutics*						
Paclitaxel^c^	228	25	5.3	20.3	-	[38]
Paclitaxel^d^	224	25	3.6	12.7	51	[37]
Paclitaxel^e^	166	25	-	15.6	-	[39]

a Patients with metastatic breast cancer, PS 0-1 on ECOG scale, received Genexol-PM (paclitaxel) at 300 mg/m^2^ *i.v.* infusion over period of 3 h, every 21 days. The median overall survival was not reached with a median follow-up of 17 months (range, 10+ to 19.8+).

b Patients with HER-2 negative metastatic breast cancer, PS 0-1 on ECOG scale, received CT-2103 (paclitaxel) at 175 mg/m^2^ *i.v.* over period of 10–20 min, every 21 days. Due to excess hypersensitivity reaction, the study was closed prior to full accrual, and the response rate was estimated.

c Patients with metastatic breast cancer, PS 0-1 on ECOG scale, paclitaxel at 175 mg/m^2^ *i.v.* infusion over period of 3 h, every 21 days.

d Patients with metastatic breast cancer, paclitaxel at 175 mg/m^2^ *i.v.* infusion over period of 3 h, for every 21 days.

e Patients with metastatic breast cancer, paclitaxel at 200 mg/m^2^ *i.v.* infusion over period of 3 h, every 21 days.

**Table 12.** Clinical outcomes of polymer paclitaxel and paclitaxel therapy in patients with non-small cell lung cancer.

**Treatment**	**N**	**Response rate**	**Median TTP**	**Median OS**	**1-year survival**	**Ref.**

		**(%)**	**(months)**	**(months)**	**(%)**	
*Polymer-paclitaxel Therapeutics*						
PG-PTX^a^	191	11	2.9	7.3	26	[46]
PG-PTX/Carboplatin^b^	199	20	3.9	7.8	31	[48]
PG-PTX^c^	427	8	2	6.9	25	[47]
Genexol-PM/Cisplatin^d^	69	38	5.8	21.7	60	[49]
*Paclitaxel Therapeutics*						
Paclitaxel/Carboplatin^e^	201	37	4.6	7.9	31	[48]
Paclitaxel/Carboplatin^f^	81	32	-	6.6	16	[42]
Paclitaxel/Carboplatin^g^	80	36	-	8.7	27	[42]
Paclitaxel/Cisplatin^h^	38	62	5.5	13.7	57	[43]
Paclitaxel/Cisplatin^i^	302	28	4.2	9.8	38	[44]
Paclitaxel/Carboplatin^j^	306	25	3	8.5	33	[44]
Paclitaxel/Carboplatin^k^	206	25	4	8.6	38	[45]

a Patients with advanced NSCLC, PS 2 on ECOG scale, received PPX (paclitaxel) at 175 mg/m^2^ every 21 days, up to 6 cycles;

b Patients with advanced NSCLC, PS 2 on ECOG scale, received PPX (paclitaxel) at 210 mg/m^2^ in combination with carboplatin (AUC = 6) every 21 days, up to 6 cycles;

c Patients with advanced NSCLC, PS 0-1 on ECOG scale, received PPX (paclitaxel) at 210 mg/m^2^ every 21 days, and at 175 mg/m^2^ every 21 days for PS 2 patients;

d Patients with advanced NSCLC, PS 0-2 on ECOG scale, received Genexol-PM (paclitaxel) at 200 mg/m^2^ (3-h *i.v.* infusion), followed by cisplatin at 60 mg/m^2^,with standard hypersensitivity reaction premedications;

e Patients with advanced NSCLC, PS 2 on ECOG scale, received paclitaxel at 225 mg/m^2^ in combination with carboplatin (AUC = 6) every 21 days, up to 6 cycles, with standard hypersensitivity reaction pre-medications;

f Patients with advanced stage IIIB or IV NSCLC, received four cycles of carboplatin at AUC of 6 and paclitaxel at 225 mg/m^2^ over 3 h every 21 days, with standard hypersensitivity reaction pre-medications;

g Patients with advanced stage IIIB or IV NSCLC, received four cycles of carboplatin at AUC of 6 and paclitaxel at 75 mg/m^2^/week for 12 weeks, with standard hypersensitivity reaction pre-medications;

h Patients with advanced stage IIIB or IV NSCLC, PS 0-2 on ECOG scale, received paclitaxel at a starting dose of 40 mg/m^2^ (1-h intravenous infusion) on days 1, 8, and 15, followed by cisplatin at a fixed dose of 80 mg/m^2^, with standard hypersensitivity reaction pre-medications. The treatment was given in a 4-week cycle;

i Patients with advanced stage IIIB or IV NSCLC, PS 0-2 on ECOG scale, received paclitaxel at 200 mg/m^2^ (3-h intravenous infusion), followed by cisplatin at a dose of 80 mg/m^2^ (30-min *i.v.* infusion), with standard hypersensitivity reaction pre-medications;

j Patients with advanced stage IIIB or IV NSCLC, PS 0-2 on ECOG scale, received paclitaxel at 200 mg/m^2^ (3-h *i.v.* infusion), followed by carboplatin at AUC of 6 (30-min *i.v.* infusion), with standard hypersensitivity reaction pre-medications;

k Patients with advanced stage IIIB or IV NSCLC, PS 0-1 on ECOG scale, received paclitaxel at 225 mg/m^2^ over 3 h with carboplatin at AUC of 6, every 21 days, with standard hypersensitivity reaction to pre-medications

**Table 13.** Clinical outcomes of polymer paclitaxel and paclitaxel therapy in patients with advanced gastric cancer.

**Treatment**	**N**	**Response rate**	**Median TTP**	**Median OS**	**1-year survival**	**Ref.**
		**(%)**	**(months)**	**(months)**	**(%)**	
*Polymer-paclitaxel Therapeutics*						
Genexol-PM/cisplatin^a^	35	46	4.9	13.8	50.2	[2]
NK105^b^	56	25	-	10.2	-	[54]
*Paclitaxel Therapeutics*						
Paclitaxel^c^	60	23	-	-	43	[53]
Paclitaxel/carboplatin^d^	27	33	-	7.5	23	[51]
Paclitaxel/cisplatin^e^	49	43	5.9	11.2	40.4	[52]

a Patients with advanced gastric cancer, PS 0-1 on ECOG scale, received Genexol® (paclitaxel) at 175 mg/m^2^ *i.v.* infusion over period of 3 h, followed by cisplatin 75 mg/m^2^ *i.v.* infusion, every 21 days, with hypersensitivity reaction to pre-medications;

b Patients with gastric cancer, received NK105 (paclitaxel) at 150 mg/m^2^ *i.v.* infusion over period of 30 min, every 21 days;

c Patients with advanced gastric cancer, PS 0-2 on ECOG scale, paclitaxel at 210 mg/m^2^ *i.v.* infusion over period of 3 h, every 21 days, with hypersensitivity reaction to pre-medications;

d Patients with advanced gastric cancer, PS 0-2 on ECOG scale, paclitaxel at 200 mg/m^2^ *i.v.* infusion over period of 3 h, followed by carboplatin AUC 5, every 21 days, with hypersensitivity reaction to pre-medications;

e Patients with advanced gastric cancer, PS 0-2 on ECOG scale, paclitaxel at 100 mg/m^2^ *i.v.* infusion over period of 1 h, followed by cisplatin 30 mg/m^2^, every 7 days, with hypersensitivity reaction to pre-medications

## Abbreviations

PTX: paclitaxel
HPMA-PTX: hydroxypropylmethacrylamide-paclitaxel conjugate
PG-PTX: poly(l-glutamic acid)-paclitaxel conjugate
PGG-PTX: poly(l-γ-glutamylglutamine) paclitaxel nano-conjugate
